# A Systematic Review of Mesenchymal Stem Cell-Derived Extracellular Vesicles: A Potential Treatment for Glioblastoma

**DOI:** 10.3390/brainsci14111058

**Published:** 2024-10-25

**Authors:** Edoardo Agosti, Sara Antonietti, Tamara Ius, Marco Maria Fontanella, Marco Zeppieri, Pier Paolo Panciani

**Affiliations:** 1 Department of Medical and Surgical Specialties, Radiological Sciences and Public Health, Division of Neurosurgery, University of Brescia, Piazza Spedali Civili 1, 25123 Brescia, Italy; edoardo_agosti@libero.it (E.A.);; 2 Neurosurgery Unit, Head-Neck and NeuroScience Department, University Hospital of Udine, p.le S. Maria della Misericordia 15, 33100 Udine, Italy; 3 Department of Ophthalmology, University Hospital of Udine, p.le S. Maria della Misericordia 15, 33100 Udine, Italy

**Keywords:** glioblastoma, molecular targets, mesenchymal stem cell, extracellular vesicle systematic reviews

## Abstract

Background: Glioblastoma (GBM) is an extremely aggressive brain tumor that has few available treatment options and a dismal prognosis. Recent research has highlighted the potential of extracellular vesicles (MSC-EVs) produced from mesenchymal stem cells as a potential treatment approach for GBM. MSC-EVs, including exosomes, microvesicles, and apoptotic bodies, perform a significant function in cellular communication and have shown promise in mediating anti-tumor effects. Purpose: This systematic literature review aims to consolidate current findings on the therapeutic potential of MSC-EVs in GBM treatment. Methods: A systematic search was conducted across major medical databases (PubMed, Web of Science, and Scopus) up to September 2024 to identify studies investigating the use of MSC-derived EVs in GBM therapy. Keywords included “extracellular vesicles”, “mesenchymal stem cells”, “targeted therapies”, “outcomes”, “adverse events”, “glioblastoma”, and “exosomes”. Inclusion criteria were studies published in English involving GBM models both in vivo and in vitro and those reporting on therapeutic outcomes of MSC-EVs. Data were extracted and analyzed based on EV characteristics, mechanisms of action, and therapeutic efficacy. Results: The review identified several key studies demonstrating the anti-tumor effects of MSC-EVs in GBM models. A total of three studies were included, focusing on studies conducted between 2021 and 2023. The review included three studies that collectively enrolled a total of 18 patients. These studies were distributed across two years, with two trials published in 2023 (66.7%) and one in 2021 (33.3%). The mean age of the participants ranged from 37 to 57 years. In terms of gender distribution, males were the predominant group in all studies. Prior to receiving MSC-EV therapy, all patients had undergone standard treatments for GBM, including surgery, chemotherapy (CT), and, in some cases, radiation therapy (RT). In all three studies, the targeted treatment involved the administration of herpes simplex virus thymidine kinase (HSVtk) gene therapy delivered to the tumor site, then 14 days of ganciclovir treatment. Outcomes across the studies indicated varying levels of efficacy for the MSC-EV-based therapy. The larger 2023 study reported fewer encouraging outcomes, with a median PFS of 11.0 months (95% CI: 8.3–13.7) and a median OS of 16.0 months (95% CI: 14.3–17.7). Adverse effects were reported in only one of the studies, the 2021 trial, where patients experienced mild-to-moderate side effects, including fever, headache, and cerebrospinal fluid leukocytosis. A total of 11 studies on preclinical trials, using in vitro and in vivo models, were included, covering publications from 2010 to 2024. The studies utilized MSCs as delivery systems for various therapeutic agents (interleukin 12, interleukin 7, doxorubicin, paclitaxel), reflecting the versatility of these cells in targeted cancer therapies. Conclusions: MSC-derived EVs represent a promising therapeutic approach for GBM, offering multiple mechanisms to inhibit tumor growth and enhance treatment efficacy. Their ability to deliver bioactive molecules and modulate the tumor microenvironment underscores their potential as a novel, cell-free therapeutic strategy. Future studies should optimize EV production and delivery methods and fully understand their long-term effects in clinical settings to harness their therapeutic potential in GBM treatment.

## 1. Introduction

Gliomas, a class of glial cell-derived brain tumors, constitute one of the most formidable obstacles in oncology. These tumors account for the majority of primary brain cancers and are associated with high morbidity and mortality. Glioblastoma (GBM), the glioma kind that is most aggressive, is notorious for its rapid progression, resistance to therapy, and poor prognosis [1]. Patients diagnosed with GBM typically face a median survival of just 12–18 months, even with aggressive treatment strategies, including surgery, radiation (RT), and chemotherapy (CT). This dismal prognosis arises from several factors inherent to the biology of gliomas, including their highly infiltrative nature, genetic heterogeneity, and ability to develop resistance to conventional treatments rapidly. Despite decades of research, no appreciable increases in overall survival (OS) have been achieved, highlighting the urgent need for novel therapeutic approaches that can effectively target the tumor’s complex microenvironment [2].

The current standard of care for GBM is concurrent CT with temozolomide (TMZ) and radiation therapy after maximal safe resection. However, even with aggressive treatment, recurrence is nearly universal. Surgical resection is often limited by the invasive nature of the tumor, which infiltrates surrounding brain tissue, making complete removal impossible without compromising critical neurological functions [2]. Furthermore, a significant barrier that hinders the effectiveness of systemic CT and limits the transport of therapeutic drugs to the tumor site is the blood–brain barrier (BBB). Radiation therapy, while effective at targeting proliferating cells, can also damage healthy brain tissue, leading to significant side effects and reduced quality of life [3]. Furthermore, GBM tumors are characterized by a high degree of inter- and intra-tumoral heterogeneity, which allows subsets of tumor cells to escape treatment and drive tumor recurrence. Finding novel therapeutic approaches that might overcome these difficulties and enhance the prognosis of glioma patients is therefore critically important.

Recent advancements in understanding cancer biology have shifted the focus towards targeting the tumor microenvironment, immune modulation, and exploiting the unique properties of cancer stem cells [4]. In this context, mesenchymal stem cells (MSCs) have gained considerable awareness due to their ability to home to sites of tissue damage and inflammation, including tumors [5]. This unique tropism for tumors and their immunomodulatory properties has made MSCs an attractive candidate for delivering therapeutic agents to cancerous tissues. Moreover, MSCs are a viable choice for therapeutic applications since they can easily be extracted and expanded in vitro from a variety of tissues, including bone marrow, adipose tissue, and umbilical cord blood [6]. However, recent studies have revealed that it is not the MSCs themselves but rather the extracellular vesicles (EVs) they secrete that play a pivotal role in mediating their therapeutic effects.

Extracellular vesicles, which are membrane-bound particles that cells spontaneously release into the extracellular environment, are mainly identified by their size and biogenesis. Examples of these particles are exosomes, microvesicles, and apoptotic bodies [7]. EVs are essential intercellular communication mediators, carrying a cargo of proteins, lipids, mRNAs, microRNAs, and other molecules that can impact the recipient cells’ activities. Mesenchymal stem cell-derived extracellular vesicles (MSC-EVs) are of particular interest due to their ability to modulate immune responses, promote tissue repair, and inhibit tumor growth. Numerous research conducted recently has shown that MSC-EVs offer therapeutic promise in a variety of illness types, including cancer, neurodegenerative disorders, and cardiovascular diseases [8].

MSC-EVs have shown promise as an innovative therapeutic approach in the context of gliomas. MSC-EVs can cross the BBB, making them ideal for delivering therapeutic cargo to brain tumors. Furthermore, MSC-EVs have been shown to exert anti-tumor effects by modulating the tumor microenvironment, inhibiting tumor cell proliferation, inducing apoptosis, and enhancing the efficacy of conventional therapies. Preclinical research has shown that MSC-EVs can slow the growth of tumors in glioma models, indicating that they may be useful as a treatment for GBM [9]. Furthermore, MSC-EVs have a low immunogenic profile, which lowers the possibility of unfavorable immune responses that are frequently connected to other cell-based treatments. As a result, there is growing interest in understanding the molecular mechanisms by which MSC-EVs exert their anti-tumor effects and exploring their potential as a targeted therapy for GBM [9].

The therapeutic effects of MSC-EVs in glioma have been examined in various experimental models, with a focus on their ability to transfer bioactive molecules to tumor cells and modulate the tumor microenvironment. Research has demonstrated that MSC-EVs are capable of delivering small RNA molecules, such as microRNAs (miRNAs), to specific signaling pathways linked to tumor growth and treatment resistance [10]. For instance, miRNAs encapsulated in MSC-EVs have been found to inhibit the expression of oncogenes and promote apoptosis in glioma cells. In addition, MSC-EVs can modulate immune responses by delivering anti-inflammatory molecules and influencing the behavior of immune cells within the tumor microenvironment [10]. MSC-EVs are a promising therapeutic option for overcoming the difficulties presented by the extremely resistant and adaptive character of gliomas because of their capacity to affect both the tumor cells and the surrounding immune cells.

Despite the promising preclinical data, the translation of MSC-EVs into clinical practice faces several challenges. One of the primary obstacles is the lack of standardized protocols for the isolation, characterization, and production of EVs for therapeutic purposes [11]. The heterogeneity of EV populations and variations in isolation techniques can lead to inconsistencies in the quality and efficacy of MSC-EV preparations. Furthermore, the mechanisms by which MSC-EVs exert their therapeutic effects in gliomas are not fully understood, and more research is needed to identify the specific molecular pathways involved. Additionally, the safety and long-term effects of MSC-EV-based therapies in humans remain to be fully elucidated. However, there is no denying that MSC-EVs have the potential to treat gliomas, and further studies will probably shed light on the processes underlying their action as well as their prospective benefits [12].

The goal of this systematic literature review is to identify the molecular pathways by which MSC-EVs exert their therapeutic effects in GBM. By analyzing and combining information from research publications, this review will seek to provide a comprehensive overview of the current understanding of MSC-EVs in the context of glioma therapy. The review will concentrate on the proteins, lipids, and nucleic acids that make up MSC-EVs’ molecular cargo and how they affect immune responses, tumor cell activity, and the tumor microenvironment. Additionally, the review will explore the mechanisms by which MSC-EVs can enhance the effectiveness of traditional treatments such as CT and RT and their potential to overcome resistance to these treatments.

## 2. Materials and Methods

### 2.1. Literature Review

The systematic review was conducted in compliance with PRISMA guidelines. Two independent researchers (E.A. and S.A.) carried out an extensive literature search across several databases, including PubMed, Ovid MEDLINE, and Scopus. The initial search took place on 31 August 2024, with an update performed on 15 September 2024. A robust search strategy was employed by combining keywords such as “glioblastomas”, “extracellular vesicles”, “mesenchymal stem cells”, “targeted therapies”, “outcomes”, and “adverse events” using both AND and OR operators. The search incorporated MeSH terms and Boolean logic: (glioblastomas OR GBM) AND (mesenchymal stem cells OR extracellular vesicles) AND (targeted therapies OR targeted treatments) AND (outcomes OR survival OR adverse events). Relevant studies were further identified by reviewing the references of the selected articles.

The inclusion criteria for selecting studies were as follows: (1) published in English; (2) in vitro, in vivo, or ex vivo studies focusing on MSC-EVs and GBM; and (3) studies that reported clinical outcomes and/or adverse events. Exclusion criteria involved: (1) editorials, case reports, case series, cohort studies, reviews, and meta-analyses; and (2) studies lacking a clear description of methods and/or results. In accordance with systematic review best practices, only primary studies were incorporated into the final analysis. All review papers that were first included have now been excluded. The current selection only emphasizes in vitro, in vivo, and clinical studies pertinent to mesenchymal stem cell-derived extracellular vesicles (MSC-EVs) in the treatment of glioblastoma.

The search results were uploaded to Endnote X9 and duplicates were removed. Each study was independently evaluated by both investigators (E.A. and S.A.) using the predefined inclusion and exclusion criteria, with any disagreements resolved by a third reviewer (P.P.P.). Finally, a full-text screening process was conducted for the articles that met all eligibility requirements.

### 2.2. Data Extraction

Each study’s details were systematically extracted, encompassing the following information: authorship, publication year, patient cohort size, previous therapeutic interventions, targeted molecular entity, studied agent, targeted therapies, clinical endpoints (progression-free survival (PFS), overall survival (OS), adverse events), and reported adverse events. Data extraction was uniformly standardized across all experiments. Data on research design, demographic characteristics, interventions, outcomes (progression-free survival, overall survival), and adverse events were rigorously gathered and organized. Every study underwent independent evaluation by two researchers, guaranteeing consistency and precision in the extraction process. In the clinical studies, mesenchymal stem cells (MSCs) were extracted from bone marrow (Study A) and adipose tissue (Study B), with one instance utilizing allogeneic MSCs (Study C). MSC-EVs were extracted using differential centrifugation and characterized based on size and surface indicators. The treatment method entailed the localized injection of MSC-EVs at the tumor location, succeeded by the administration of ganciclovir.

### 2.3. Outcomes

The primary outcomes were progression-free survival (PFS), OS, and adverse events. Subsidiary outcomes encompassed evaluating clinical results and identifying adverse events associated with these interventions.

### 2.4. Risk of Bias Assessment

**Figure 1:**
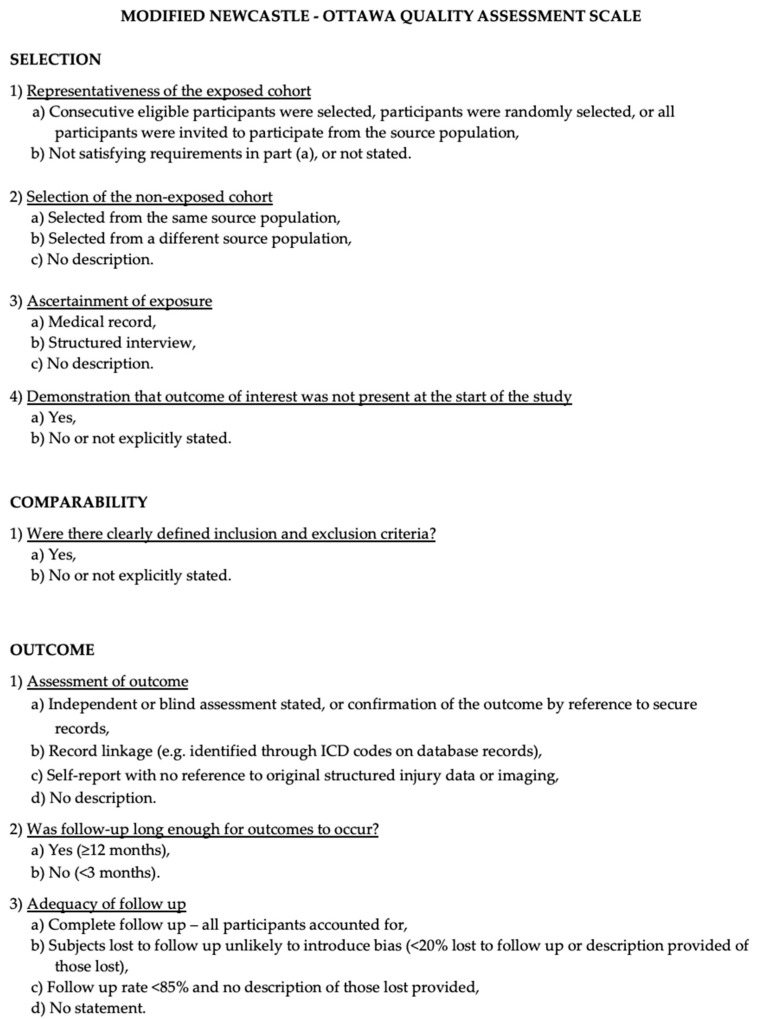
The modified NOS.

The evaluation of study quality was conducted using the Newcastle–Ottawa Scale (NOS), which appraised the included studies based on selection criteria, comparability, and outcome assessment. Quality appraisal involved assessing the abovementioned aspects, with an optimal score of 9. Elevated scores denoted superior study quality, with studies garnering 7 or more points classified as high-quality. Quality assessment was independently conducted by two authors (E.A. and P.P.P.), and the third author resolved any disparities through re-examination (Figure 1).

### 2.5. Statistical Analysis

The descriptive statistics provided included ranges and percentages. All statistical studies used the R statistical software, version 3.4.1 (http://www.r-project.org, accessed on 15 September 2024).

## 3. Results

### 3.1. Literature Review

**Figure 2:**
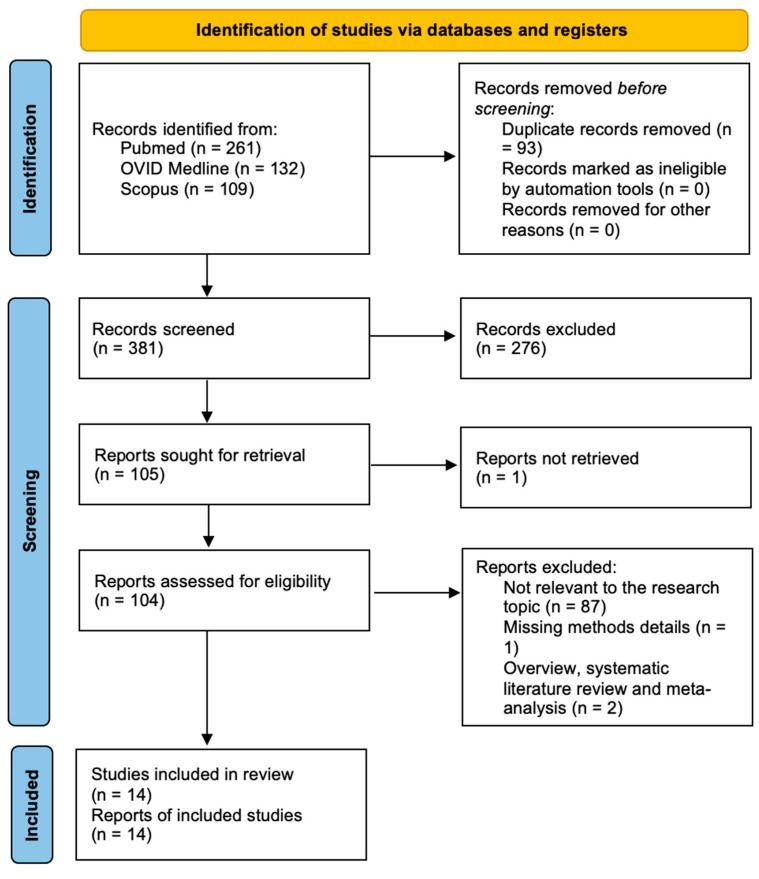
PRISMA flow chart.

After duplicates were eliminated, 381 papers in total were found. A total of 105 articles were found for full-text analysis after title and abstract analysis. Eligibility was assessed for 104 articles and ascertained for 14 articles. The following criteria led to the exclusion of the remaining 90 articles: 87 publications unrelated to the study issue, two articles that are systematic literature reviews or meta-analyses, and one article that does not provide methodological or result information. For each of the patient groups under consideration, at least one or more outcome measures were available for all of the studies that were part of the analysis. This systematic review was not registered in any database, including PROSPERO. While registration enhances transparency, PRISMA criteria were scrupulously followed to ensure methodological rigor. The PRISMA statement’s flow chart is seen in Figure 2.

**Figure A1:**
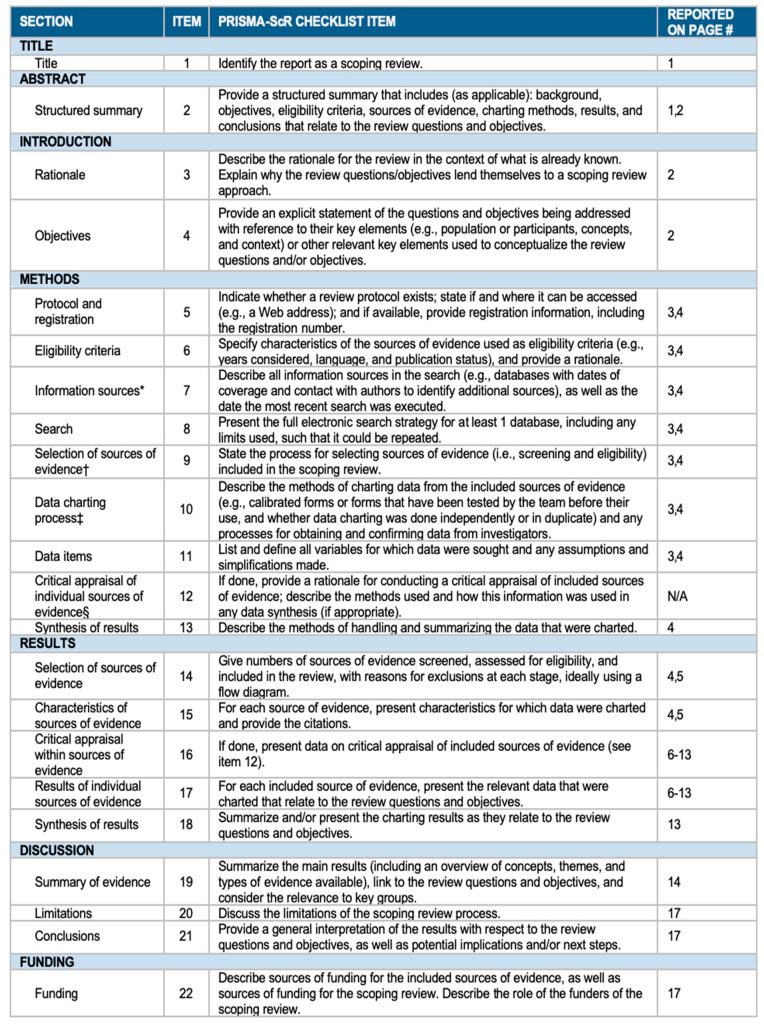
The PRISMA-ScR checklist. Abbreviations: JBI = Joanna Briggs Institute; PRISMA-ScR = Preferred Reporting Items for Systematic Reviews and Meta-Analyses extension for Scoping Reviews. * Where sources of evidence (see second footnote) are compiled from, such as bibliographic databases, social media platforms, and Web sites. † A more inclusive/heterogeneous term used to account for the different types of evidence or data sources (e.g., quantitative and/or qualitative research, expert opinion, and policy documents) that may be eligible in a scoping review as opposed to studies only. This is not to be confused with information sources (see first footnote). ‡ The frameworks by Arksey and O’Malley (6) and Levac and colleagues (7) and the JBI guidance (4, 5) refer to the process of data extraction in a scoping review as data charting. § The process of systematically examining research evidence to assess its validity, results, and relevance before using it to inform a decision. This term is used for items 12 and 19 instead of “risk of bias” (which is more applicable to systematic reviews of interventions) to include and acknowledge the various sources of evidence that may be used in a scoping review (e.g., quantitative and/or qualitative research, expert opinion, and policy document).

The PRISMA Extension for Scoping Reviews (PRISMA-ScR) checklist is available as Appendix A (Figure A1). 

### 3.2. Data Analysis

Table 1 and Table 2 present a summary of the included studies reporting on targeted therapies for skull base glioblastomas, respectively, for clinical studies and preclinical studies.

#### 3.2.1. Clinical Studies

Three studies were included, focusing on studies conducted between 2021 and 2023. The review included three studies that collectively enrolled 18 patients. These studies were distributed across two years, with two trials published in 2023 (66.7%) and one in 2021 (33.3%). This relatively recent period of publication highlights the growing interest in MSC-EV-based therapies for GBM, especially in recent years [13,14,15].

In terms of patient demographics, the studies included a range of patient populations, with the number of participants per study varying from a single patient in one case study to 12 patients in the largest 2023 trial (B). Another study conducted in 2021 (A) included five patients. The mean age of the participants ranged from 37 to 57 years, indicating that the trials spanned a broad adult population. In the 2021 study, the mean patient age was 46 years, with a range from 32 to 62 years. In the two 2023 studies (B,C), one reported a mean age of 57 years, while the case study involved a single 37-year-old patient [13,14,15].

In terms of gender distribution, males were the predominant group in all studies. The 2021 study had 80% male participants (4 out of 5), while the largest 2023 study had a male participation rate of 66.7% (8 out of 12 patients). In the single-patient case study from 2023, the participant was male. Overall, 72.2% of the patients across all the trials were male, reflecting a significant gender imbalance that may warrant further exploration in future research to assess the potential impact of sex differences on treatment outcomes [13,14,15].

Prior to receiving MSC-EV therapy, all patients had undergone standard treatments for GBM, including surgery, CT, and, in some cases, RT. These standard interventions were typically administered to manage the tumor before the experimental therapy was applied, indicating that MSC-EV therapy was primarily investigated as a follow-up or salvage therapy for patients who had already been treated with conventional approaches. This is consistent with the challenging nature of GBM, where recurrence after standard treatment is common and necessitates the exploration of novel therapeutic strategies.

In all three studies, the targeted treatment involved the administration of HSVtk gene therapy delivered to the tumor site, followed by ganciclovir administration for 14 days. This gene therapy approach, facilitated by MSC-EVs as the delivery vehicle, aimed to selectively target GBM cells, leveraging the vesicles’ ability to cross the BBB and reach tumor foci. The use of MSC-EVs as a carrier for gene therapy provides a promising avenue for targeted GBM treatment, offering potential advantages over conventional therapies, which often face challenges in effectively reaching the tumor due to the presence of the BBB.

The follow-up periods for these trials varied. In one of the 2023 studies (B), the mean follow-up duration was reported as 16 months, while the single-patient case study (C) from the same year had a 12-month follow-up. Unfortunately, follow-up data for the 2021 study were not provided. These follow-up durations allowed researchers to monitor the patients’ PFS and OS, which were the primary outcomes of interest in these trials.

Outcomes across the studies indicated varying levels of efficacy for the MSC-EV-based therapy. Survival outcomes differed across the research included. The effects of MSC-EV treatments on progression-free survival (PFS) and overall survival (OS) yielded inconsistent outcomes, with a mean PFS of 21 months in one research and a median OS of 16 months in another. Additional subgroup analysis is required to evaluate the impact of patient variables, such as age, sex, and previous treatments, on these results. In the 2021 study, patients experienced a relatively favorable outcome, with a mean PFS of 21 months and a mean OS of 29 months. Furthermore, at one year, 60% of patients remained progression-free and 100% had survived, suggesting that the MSC-EV therapy may have substantially impacted this small patient cohort. In contrast, the larger 2023 study reported fewer encouraging outcomes, with a median PFS of 11.0 months (95% CI: 8.3–13.7) and a median OS of 16.0 months (95% CI: 14.3–17.7). In this study, 11 out of 12 patients exhibited progressive disease (PD), indicating that the therapy was less effective in controlling tumor progression. The single-patient case study from 2023 also reported PD despite the treatment, suggesting that the effectiveness of MSC-EV therapy may be variable and dependent on individual patient factors.

Adverse effects were reported in only one of the studies, the 2021 trial, where patients experienced mild-to-moderate side effects, including fever, headache, and cerebrospinal fluid leukocytosis. These side effects were manageable and did not appear to result in serious complications. However, it is notable that the other two studies from 2023 did not report any adverse effects, either due to a lack of significant side effects or possible underreporting. Future studies will need to further clarify the safety profile of MSC-EV therapy (Table 1).

#### 3.2.2. Preclinical Studies

Eleven studies in all were included, covering publications from 2010 to 2024. The timeframe indicates an increasing interest in this therapeutic approach, with most studies published between 2015 and 2020; specifically, the earliest study was from 2010, and the most recent was from 2024. This suggests that the development of MSC-based therapies for GBM has been gaining momentum in recent years, driven by the ability of MSCs to transport a variety of therapeutic substances efficiently.

Most of the included studies were preclinical, using in vitro and in vivo models, rather than clinical trials involving human subjects. These studies explored the potential of MSCs and MSC-EVs in delivering therapeutic agents to GBM cells, aiming to improve OS and PFS. Since these trials have not yet advanced to Phases I–III clinical testing, the focus remained largely on understanding the methods of action and testing the efficacy of MSC-based treatments in animal models and under laboratory conditions. No patient data were available, underscoring the experimental nature of this research and highlighting that further investigation is needed before MSC-EV therapies can be tested on a broader clinical scale.

The studies utilized MSCs as delivery systems for various therapeutic agents, reflecting the versatility of these cells in targeted cancer therapies. For instance, some studies focused on immunomodulation, using MSCs engineered to express immune-stimulating cytokines such as IL-12 and IL-7. These agents were shown to alter the tumor’s immunosuppressive microenvironment and promote anti-cancer immune responses. Chemotherapy delivery was another significant focus, with several studies investigating paclitaxel (PTX)-loaded MSCs, showing that these cells could target GBM cells specifically while sparing healthy tissue. Studies using doxorubicin (DOX)-polymer conjugates similarly demonstrated enhanced tumor penetration and cytotoxicity when delivered via MSCs.

Gene therapy approaches were also well-represented, with researchers exploring the use of MSC-HSV-TK, which induces tumor cell death through a suicide gene system, and MSC tumor necrosis factor (TNF)-related apoptosis-inducing ligand (TRAIL), where MSCs delivered apoptosis-inducing ligands to GBM cells. In several of these studies, MSC-based delivery systems were shown to significantly improve survival and delay tumor progression in preclinical animal models, suggesting their potential to enhance treatment outcomes for GBM patients in the future.

Some studies explored the use of MSCs as viral carriers for oncolytic therapy, such as the 2021 study employing patient-derived bone marrow-derived human mesenchymal stem cells loaded with Delta-24-RGD (PD-BM-MSC-D24), which demonstrated that MSCs could efficiently deliver oncolytic viruses to GBM cells. Other research investigated cytokine delivery systems, like the 2020 study where MSCs overexpressing beta-interferon (IFNβ) were employed to treat GBM, showing a strong therapeutic effect in animal models.

In terms of outcomes, while none of the studies directly involved human patients or measured clinical endpoints such as OS or PFS, significant improvements in tumor progression and survival were observed in animal models. Across multiple studies, MSC-based therapies led to extended survival and tumor growth reduction, with some demonstrating prolonged progression-free periods. These results imply that MSC-EVs, which deliver therapeutic chemicals more accurately and efficiently than conventional techniques, may be essential to the development of new GBM treatments.

Chemotherapeutic delivery, specifically using paclitaxel (PTX)-loaded MSCs, was the most frequently reported therapeutic strategy, accounting for 18.2% of the studies. Gene therapies, including suicide gene therapy and apoptosis-inducing ligands, followed closely behind. Immunomodulatory treatments using cytokines like IL-12 and IFNβ were less common but demonstrated significant potential, particularly in creating an immune-favorable tumor microenvironment. Viral delivery via MSCs was reported in only a small subset of studies, but these findings were promising for future oncolytic virus therapies targeting GBM (Table 2).

## 4. Discussion

Despite improvements in surgery, CT, and RT, the prognosis for glioblastoma, a very aggressive and deadly form of brain cancer, is infamously bad.

The emergence of MSC-EVs as therapeutic agents offers a fascinating new direction in targeted GBM treatment [7]. This systematic review explored several preclinical studies on MSC-EVs and MSC-based therapies for GBM. The findings indicate that MSCs hold significant promise as vehicles for delivering various anti-cancer agents directly to the tumor site, potentially overcoming the limitations of the BBB and enhancing tumor cell specificity. However, while preclinical results are encouraging, significant challenges remain before MSC-EV therapies can be translated into clinical practice. This section discusses the molecular mechanisms, targeted therapies, broader implications based on the review findings, and relevant comparisons to the existing literature [27].

### 4.1. Molecular Mechanisms of MSC-EVs in GBM Therapy

Because of their capacity to permeate the blood–brain barrier and alter the tumor microenvironment, MSC-EVs have a number of benefits when used as a delivery system for GBM treatment. This review’s several studies showed how MSCs might be modified to produce and transfer therapeutic compounds to GBM cells, causing tumor cytotoxicity and modifying immune responses [28].

In the study by Mohme et al. [16], MSCs were used to co-express interleukin 12 and 17 (IL-12 and IL-7), cytokines with known roles in promoting anti-cancer immune responses. These cytokines modified the immunosuppressive environment of GBM, allowing for greater immune system recognition of tumor cells. This study echoes the findings of Agosti et al. [29], who observed similar immunomodulatory effects in GBM models. Immunomodulation represents one of the most promising mechanisms through which MSC-EVs exert therapeutic effects, as the immune evasion characteristics of GBM are well-documented barriers to effective treatment [29].

The 2014 study by Zhang et al. [17] also explored the use of DOX-polymer conjugates (PPCD), which were modified by RGD peptides for targeted delivery. This study reported that MSCs loaded with RGD-PPCD displayed enhanced tumor penetration and increased cell death compared to MSCs loaded with DOX alone [17]. These findings are consistent with the broader literature on MSC-based drug delivery systems, where ligands like RGD have been shown to enhance the selectivity and effectiveness of MSC-EV therapies. In comparison, Bryukhovetskty et al. [18] demonstrated the effectiveness of combining temozolomide (TMZ) with MSC transplantation, which dramatically increased survival in a rat model of glioma, highlighting the possibilities for MSC-EVs to deliver established chemotherapeutic agents more effectively [18].

### 4.2. Targeted Therapies Using MSC-EVs

The prospects for MSC-EVs to deliver targeted therapies was a central theme across many studies included in this review. Multiple studies investigated the ability of MSCs to deliver chemotherapeutic agents, gene therapies, and oncolytic viruses. For instance, Malik et al. [22] utilized PEI-PLL copolymers to engineer MSCs with HSV-TK and TRAIL genes, a combination that induced significant tumor regression both in vitro and in vivo. This study supports the broader literature, including the work of Maeda et al. [30], who found that gene therapy approaches using MSC-EVs could overcome traditional CT resistance by delivering genetic material directly into GBM cells, bypassing mechanisms of drug efflux [30].

Another promising approach involved the use of MSCs as vehicles for delivering PTX, as demonstrated in studies by Pacioni et al. [19,20,21]. These studies confirmed that PTX-loaded MSCs could specifically target GBM cells while sparing healthy brain tissue, emphasizing the ability of MSCs to home to the tumor microenvironment. This selective targeting capability is a crucial advantage of MSC-based therapies over conventional CT, where systemic toxicity limits the dose that can be safely administered. Similar results were observed by Jiang et al. [20], where TRAIL-overexpressing adipose-derived MSCs in a GBM model improved survival by slowing tumor growth [20].

A unique approach was presented by Shimizu et al. [25], who used MSC-D24 as oncolytic viral carriers, showing that these cells could efficiently deliver viral particles to the tumor. This aligns with findings from Xu et al. [24], who employed a non-viral technique to transiently engineer MSCs to express therapeutic transgenes, presenting a scalable method for treating TMZ-resistant GBM. Both studies highlight the potential for MSCs to enhance oncolytic virotherapy. This approach has seen limited success in clinical trials but could benefit from the tumor-homing properties of MSC-EVs [24,25,31,32,33,34].

### 4.3. Comparative Analysis with the Existing Literature

The findings of the studies that were examined in this systematic analysis are in good agreement with previous studies on the application of MSCs and MSC-EVs in cancer treatment. For example, Pacioni et al. [19] and Zhang et al. [17] both highlight the ability of MSCs to deliver chemotherapeutic agents directly to tumor sites, reducing systemic toxicity—a benefit consistently noted in recent studies on MSC-based drug delivery [29]. The emphasis on immunomodulation, seen in Mohme et al. [16] and supported by studies like those of Agosti et al. [29] and Maeda et al. [30], suggests that MSCs may play a dual role in GBM treatment by both delivering therapeutic agents and modulating the immune response.

Gene therapy approaches, such as those described by Malik et al. [22] and Xu En Tu et al. [24], also reflect broader trends in GBM research, where the focus has shifted towards exploiting the tumor microenvironment and targeting genetic vulnerabilities. However, the delivery of therapeutic agents via MSC-EVs offers the additional advantage of increased targeting precision, as MSCs can cross the BBB and preferentially accumulate in tumor tissue, an advantage noted by both Shimizu et al. [25] and Jiang et al. [20]

### 4.4. Challenges, Considerations, and Future Developments

In spite of the encouraging results, there are still a number of obstacles to overcome before MSC-EV treatments can be used in patients. One of the primary obstacles is the lack of large-scale clinical trials. Most studies to date have been preclinical, utilizing animal models and cell lines to assess the efficacy of MSC-EV therapies. The absence of Phase I–III clinical trials makes it difficult to assess the safety and potential side effects of these treatments in humans, particularly given the complex nature of MSC-EV biology and the potential for off-target effects [35,36]. The clinical studies reviewed have a low sample size (n = 18), which constrains the statistical power and generalizability of the results. The limited cohort may create bias and compromise the validity of conclusions about progression-free survival (PFS) and overall survival (OS). Extensive, more thorough studies are required to corroborate these preliminary results. The clinical studies examined exhibited diverse outcomes. Study A indicated an enhancement in PFS of up to 21 months and OS of up to 29 months, whilst Study B showed an OS of up to 16 months and a PFS of up to 11 months. Study C indicated an OS of up to 17 months and a PFS of up to 9 months. Notwithstanding the limited sample sizes, these trials indicate the promise of MSC-EV treatment in prolonging survival among patients with recurrent GBM [35,36]. Another critical issue is the standardization of MSC isolation, EV production, and delivery methods. Variability in the production and characterization of MSC-EVs could result in inconsistent therapeutic effects, as Park et al. [26] noted the need for scalable reproducible techniques to generate engineered MSCs for clinical use [26]. Additionally, the immunogenicity of MSC-EVs, though generally considered low, must be thoroughly evaluated before these therapies can be widely adopted. MSC-EVs exhibit considerable potential as an integral component of a multimodal therapeutic approach for glioblastoma, especially when utilized in conjunction with chemotherapy, radiation, and immunotherapy. The difficulty in increasing MSC-EV production resides in maintaining uniformity in vesicle composition, yield, and functionality. Standardizing production techniques and mitigating possible immune responses are essential stages for the therapeutic deployment of MSC-EVs.

Subsequent advancements ought to concentrate on incorporating MSC-EV treatments into multimodal therapeutic strategies. Combining MSC-based therapies with existing modalities like CT, RT, or immune checkpoint inhibitors (as demonstrated by Shimizu et al. [25]) holds the potential to enhance treatment efficacy and overcome some of the resistance mechanisms that have limited the success of traditional therapies. Furthermore, identifying biomarkers that predict patient responses to MSC-EV therapies could pave the way for personalized treatment approaches, making sure that clinical trial participants who are most likely to benefit from these therapies are chosen [37,38,39,40].

## 5. Conclusions

The substantial potential of MSC-EVs as GBM treatment agents is highlighted by the studies included in this systematic analysis. The ability of MSCs to deliver a wide range of therapeutic agents—chemotherapeutic drugs, gene therapies, immunomodulators, and oncolytic viruses—positions them as a versatile tool in the fight against this deadly cancer. However, the preclinical nature of most studies highlights the need for further research and clinical trials to fully assess the safety, efficacy, and scalability of MSC-EV therapies. As we move towards a more personalized approach to cancer treatment, the integration of MSC-EV therapies into multimodal treatment strategies holds promise for improving outcomes for GBM patients. To guarantee that these therapies are successfully incorporated into clinical procedures, issues with potential immunogenicity, patient selection, and production uniformity must be resolved.

## Figures and Tables

**Table 1:** Summary of clinical studies included in the systematic literature review reporting on the role of MSC-EVs as a therapeutic for GBM.

Author, Year	Oraee Yazdani, 2021 [13]	Oraee Yazdani, 2023 [14]	Oraee Yazdani, 2023 [15]
Patients (N)	5	12	1
Age (mean, range)	46, (32, 62)	57	37
Sex M (F/M ratio)	4, 80.0%	8, 66.7%	1, 100%
Prior treatment	Surgery, CT, RT	Surgery, CT	Surgery
MSCs targeted treatment agent	HSVtk gene to the tumor’s frontal focus, and then take ganciclovir for 14 days.	HSVtk gene to the tumor’s frontal focus, and then take ganciclovir for 14 days.	HSVtk gene to the tumor’s frontal focus, and then take ganciclovir for 14 days.
Next treatment	N/A	N/A	N/A
Mean Follow-up (months)	N/A	16	12
Outcome	PFS 21 mos OS 29 mos 1-year PFS: 60% 1-year OS: 100%	PD (11 pts) OS: 16.0 mos PFS: 11.0 mos	PD PFS: 9 mos OS: 17 mos
Adverse effects	Mild-to-moderate fever, headache, and cerebrospinal fluid leukocytosis	N/A	N/A

Abbreviations: CT: chemotherapy; RT: radiation therapy; N/A = not applicable; HSVtk: herpes simplex virus thymidine kinase; M: male; OS: overall survival; PFS: progression-free survival; PD: progressive disease.

**Table 2:** Summary of preclinical studies included in the systematic literature review reporting on the role of MSC-EVs as a therapeutic for GBM.

Author, Year	Study Type	MSC Treatment	Study Purpose	Results
Zhang et al., 2014 [16]	In vitro, in vivo (ICR mice with orthotopic intracranial C6 glioma)	doxorubicin (DOX)-polymer conjugates (PPCD) modified by RGD (RGD-PPCD)	The study compared the anti-tumor properties of MSCs loaded with RGD-/PPCD (MSCsRGD-/PPCD) compared to their corresponding RGD-/PPCD and MSCsDOX.	Compared to MSCsDOX, MSCsRGD-/PPCD showed greater penetration and increased tumor cell death.
Bryukhovetskty et al., 2015 [17]	In vivo (C6 glioma Wistar rat models)	Multipotent mesenchymal stromal cells and TMZ	The study investigates the results of MMSC transplantation during chemotherapy for a C6 glioma model in rats.	The survival of experimental animals treated with MMSC transplantation and temozolomide treatment was dramatically increased compared to animals treated with temozolomide alone.
Pacioni et al., 2015 [18]	In vivo (xenografted U87MG cells)	PTX loaded MSCs	The GBM model is employed to ascertain whether PTX-MSCs continue to exhibit a preference for the tumor cells and describe the cytotoxic harm brought on by MSC-driven PTX release in the tumor microenvironment.	Since PTX may selectively kill GBM cells while avoiding negative effects on normal tissue, MSCs are well adapted for the administration of anti-neoplastic medications in the brain.
Jiang et al., 2016 [19]	In vivo (GBM xenograft mice)	hADSCs overexpressing TRAIL	hADSCs produced from fat tissue could be a better option than stem cell-based cancer gene therapy. Several biological medications, such as prodrugs, chemotherapeutic agents, and genetic signals, may be administered by utilizing stem cells as drug delivery vehicles.	hADSCs overexpressing TRAIL slowed the growth of GBM, increased survival, and decreased the number of microsatellites.
Pacioni et al., 2017 [20]	In vivo (orthotopic xenografts of U87MG cells)	PTX-loaded hMSCs	The capacity of MSCs to release bioactive chemicals makes them an appealing option for cell-based cancer therapy.	hMSCs have a therapeutic potential in GBM brain xenografts that are also expressed negatively against the GSC population.
Malik et al., 2018 [21]	In vitro, in vivo (C6 glioma cells rat models)	PEI-PLL copolymer to generate genetically engineered MSCs with suicidal genes, HSV-TK, and TRAIL.	Viral vectors that can cause oncogenicity and restrict the use of MSCs in clinical trials have been used to modify the cells for cancer therapy genetically. Non-viral agents, such as PEI-PLL, were employed in this investigation.	An intratumoral injection of polymer-double-transfected MSCs combined with prodrug ganciclovir can significantly enhance the therapeutic response in vitro and in vivo.
Mao et al., 2020 [22]	In vivo (animals bearing orthotopic gliomas)	IFNβ and FTH overexpressed MSCs (IFNβ-FTH-MSCs)	This work used in vivo MRI tracking to investigate whether MSCs may be employed as cellular carriers to deliver IFNβ locally for glioma therapy.	MSCs can be employed as IFNβ cellular carriers to treat malignant glioma effectively.
Tu et al., 2020 [23]	In vitro, in vivo (glioma cells nude rats models)	CD::UPRT::GFP-expressing MSCs	The purpose of this work was to find a simple, highly effective, and scalable non-viral technique for transiently engineering MSCs to express a fused transgene over an extended period of time at abnormally high levels.	The effective non-viral approach may allow for the scalable translation of therapeutically altered MSC in the management of GBM that is resistant to TMZ.
Mohme et al., 2020 [24]	In vivo (orthotopic syngeneic glioma model in C57BL/6 mice)	MSCs to co-express high levels of IL-12 and IL-7 (MSCIL7)	Motile MSC-based local immunomodulation potential was validated to stimulate an anticancer immune response and overcome the immunosuppressive glioblastoma microenvironment.	It is possible to effectively modify the immunosuppressive milieu in glioblastoma through local MSC-based immunomodulation.
Shimizu et al., 2021 [25]	In vitro, in vivo (U87MG cells nude mice models)	PD-BM-MSC-D24	Human mesenchymal stem cells generated from bone marrow and taken from healthy donors were studied for their potential as viral carriers.	BM-hMSCs can be obtained from patients who have had marrow-toxic chemotherapy. These cells, known as PD-BM-hMSCs, are efficient oncolytic viral carriers.
Park et al., 2024 [26]	In vivo (glioma cells C57BL/6 mice models)	IL-12-secreting mesenchymal stem cells	The therapeutic effects of anti-PD-1, MSC_IL-12, and their combination against glioblastoma were assessed using IL-12-secreting mesenchymal stem cells that were generated with glioma tropism.	Long-term treatment responses are observed with anti-PD-1 and MSC-IL-12 monotherapies; their combination increases anticancer efficacy against glioblastoma by generating an immune-favorable tumor microenvironment.

Abbreviations: BM-hMSCs = bone marrow-derived human mesenchymal stem cells; CD::UPRT::GFP = yeast cytosine deaminase::uracil phosphoribosyl-transferase::green fluorescent protein; hADSCs = polymeric nanoparticle-engineered human adipose-derived stem cells; FTH = ferritin heavy chain; IL-12 = interleukin 12; IFNβ = beta-interferon; MSCs = mesenchymal stem cells; MSCsDOX = MSCs loaded with DOX; PD-BM-hMSCs = patient-derived BM-hMSCs; PD-BM-MSC-D2 = PD-BM-hMSCs loaded with Delta-24-RGD; PEI-PLL = polylysine-modified polyethylenimine; PTX = paclitaxel; TMZ = temozolomide; TRAIL = tumor necrosis factor-related apoptosis-inducing ligand.

## Data Availability

Data are available in a publicly accessible repository. No new data were created.
